# Supplementary material for: NH2-Functionalized Magnetic Nanoparticles for the N-Glycomic Analysis of Patients with Multiple Sclerosis
Source: Int J Mol Sci. 2022 Aug 13;23(16):9095. doi: 10.3390/ijms23169095 (PMC9409089; doi:10.3390/ijms23169095)
Supplement: Supplementary file 1 [file ijms-23-09095-s001.zip › ijms-1819494-supplementary.pdf]

Supplementary Material of the manuscript entitled NH_2-Functionalized Magnetic Nanoparticles for The N-glycomic Analysis of Patients with Multiple Sclerosis

Dalma Dojcsák [1]. Ágnes Ilosvai [2]. László Vanyorek [2]. Ibolya Gilányi [3]. Csaba Oláh [3]. László Horváth [3] and Csaba Váradi [1, *]

[1] Advanced Materials and Intelligent Technologies Higher Education and Industrial Cooperation Centre. University of Miskolc. Hungary 3515;
[2] Institute of Chemistry. Faculty of Materials Science and Engineering. University of Miskolc. Hungary 3515;
[3] Borsod-Abaúj-Zemplén County Center Hospital and University Teaching Hospital. Miskolc. Hungary 3526
* Correspondence: kemcsv@uni-miskolc.hu; Tel.: +36308947730

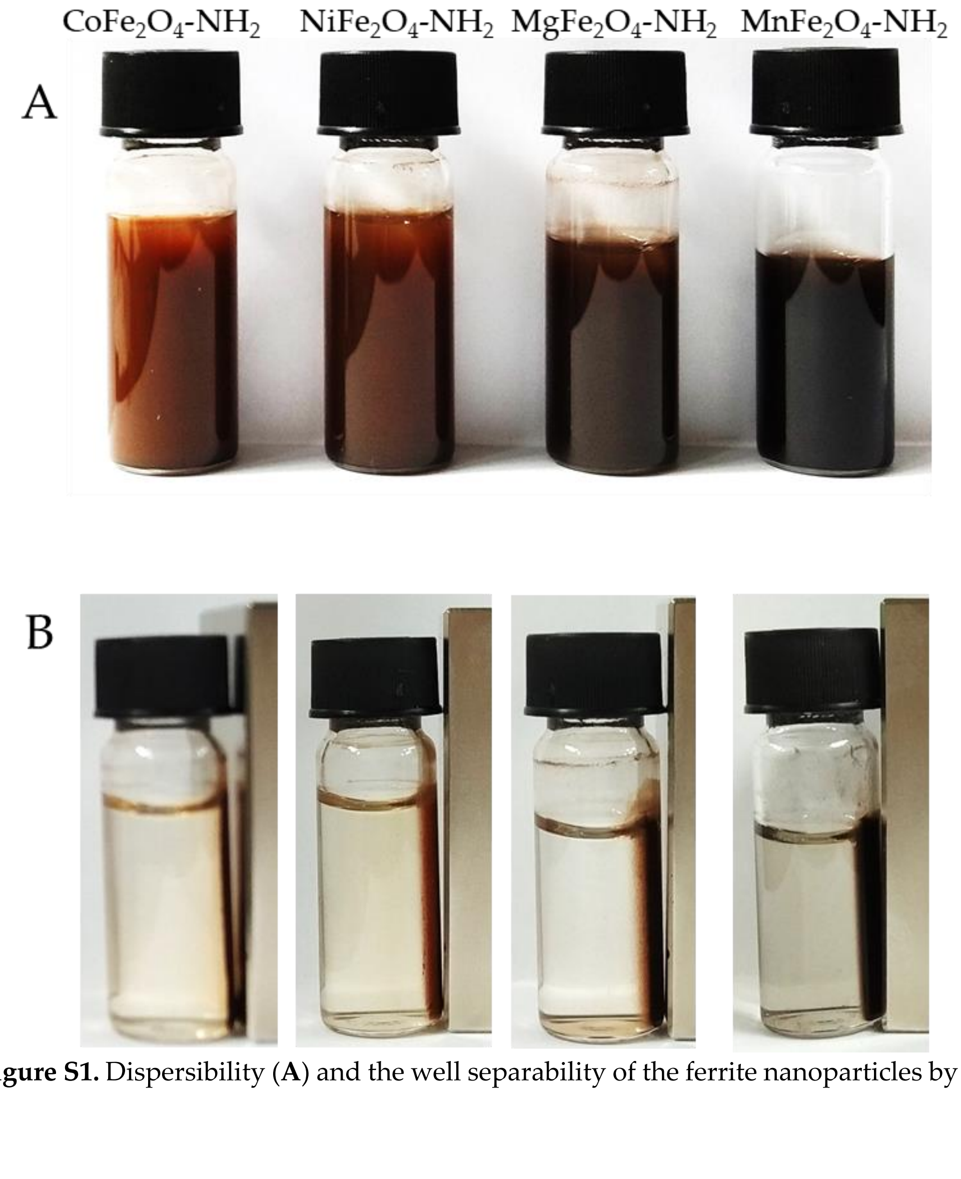

Supplementary Figure S1. Dispersibility (**A**) and the well separability of the ferrite nanoparticles by magnetic field (**B**).

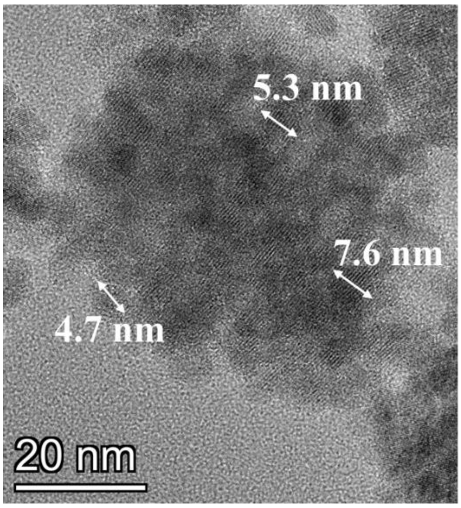

Supplementary Figure S2. HRTEM picture $MnFe_2O_4$ nanosphere and the forming nanoparticles.

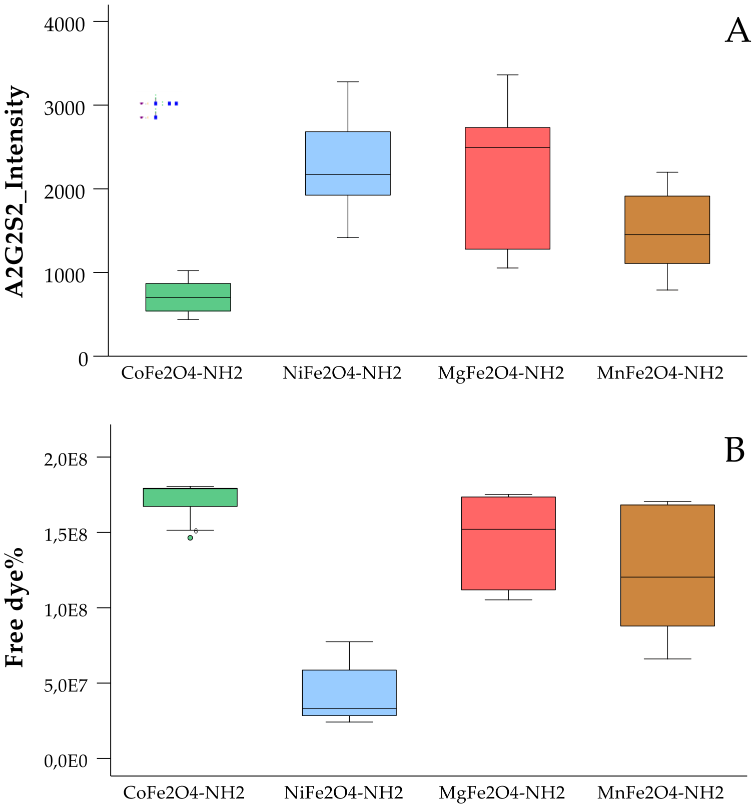

Supplementary Figure S3. Signal intensity of purified glycan structure (**A**) and free procainamide (**B**) and using different NH_2-Functionalized Magnetic Nanoparticle.

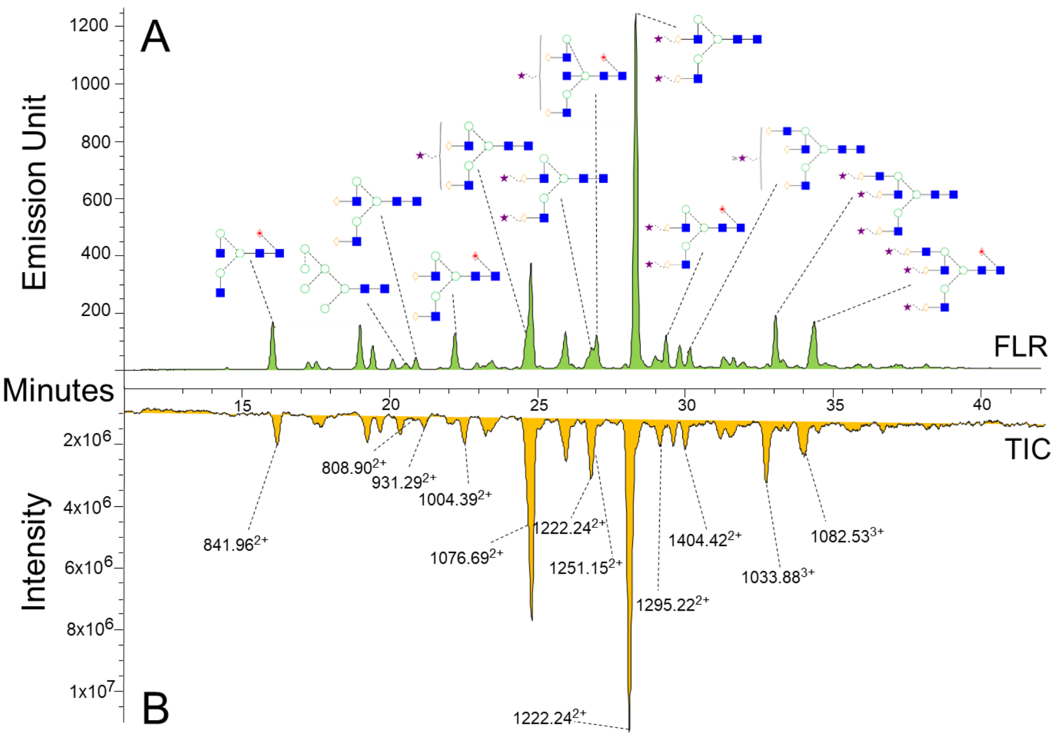

Supplementary Figure S4. Profile of human serum N-glycome analyzed by hydrophilic interaction liquid chromatography with fluorescence (**A**) and mass-spectrometric detection (**B**).

Supplementary Table S1. Calculation of variation coefficient throughout the quantified 43 peaks of HILIC chromatograms.

	Mean	Standard deviation	CV
Peak 1	3.18	0.07	2.14
Peak 2	0.81	0.03	4.04
Peak 3	0.84	0.02	2.38
Peak 4	4.02	0.07	1.71
Peak 5	2.26	0.04	1.93
Peak 6	1.20	0.02	1.84
Peak 7	0.23	0.02	10.00
Peak 8	1.07	0.06	5.16
Peak 9	1.06	0.03	2.91
Peak 10	4.11	0.05	1.17
Peak 11	1.06	0.01	1.29
Peak 12	1.33	0.01	0.81
Peak 13	2.74	0.05	1.86
Peak 14	8.47	0.05	0.60
Peak 15	1.75	0.22	12.82
Peak 16	3.87	0.19	4.88
Peak 17	0.55	0.01	1.95

Peak 18	1.46	0.07	4.88
Peak 19	3.86	0.07	1.72
Peak 20	0.38	0.00	0.53
Peak 21	28.07	0.20	0.71
Peak 22	0.36	0.04	12.39
Peak 23	1.20	0.06	5.12
Peak 24	3.91	0.07	1.83
Peak 25	1.72	0.01	0.72
Peak 26	1.99	0.01	0.69
Peak 27	1.20	0.03	2.70
Peak 28	0.54	0.03	5.76
Peak 29	0.56	0.00	0.72
Peak 30	0.80	0.01	0.92
Peak 31	0.18	0.03	14.04
Peak 32	5.93	0.11	1.78
Peak 33	0.83	0.01	1.68
Peak 34	0.59	0.02	2.96
Peak 35	1.84	0.05	2.70
Peak 36	2.21	0.04	1.93
Peak 37	1.16	0.02	1.88
Peak 38	0.61	0.01	1.56
Peak 39	0.61	0.02	3.84
Peak 40	0.16	0.01	7.80
Peak 41	0.61	0.03	5.60
Peak 42	0.29	0.01	3.94
Peak 43	0.37	0.02	5.50
Mean		0.05	3.52

Supplementary Table S2. Significant N-glycome alterations between multiple sclerosis patients and healthy controls.

			Healthy_Female		Healthy_Male		Sclerosis_Female		Sclerosis_Male		
Retention time	*Structure*	*m/z*	*Area % average*	*Area % std*	*Area % average*	*Area % std*	*Area % average*	*Area % std*	*Area % average*	*Area % std*	*Significance level (p-value)*
16.07	FA2	842.11	3.41	1.13	3.88	1.26	2.83	0.98	3.67	1.40	0.04
17.29	M5	727.93	1.00	0.26	0.97	0.20	1.15	0.33	0.87	0.16	0.04
20.64	M6	809.09	0.98	0.30	0.93	0.20	1.22	0.39	0.89	0.24	0.02
20.94	A2G2	931.18	0.96	0.14	0.91	0.14	1.12	0.13	1.02	0.08	0.01
22.98	FA2BG2	1105.83	0.76	0.18	0.71	0.18	1.10	0.24	0.76	0.18	0.01
23.43	FA2BFG1	1097.40	1.10	0.19	1.16	0.16	0.91	0.18	1.00	0.25	0.01
24.57	A2G2S1	1076.77	2.78	0.24	2.77	0.32	3.21	0.44	3.09	0.66	0.01
25.89	FA2G2S1	1149.74	3.49	0.81	3.46	0.65	2.95	0.69	3.00	0.98	0.05
26.78	A2G2S2	1222.53	2.66	0.90	2.61	0.79	1.72	0.41	1.82	0.62	0.01
26.90	FA2BG2S1	1251.62	3.21	0.72	2.85	0.58	4.07	0.96	3.42	0.58	0.01
28.45	A2G2S2	1221.94	0.56	0.08	0.65	0.25	0.39	0.10	0.46	0.05	0.01
28.98	FA2G2S2	1295.36	1.50	0.22	1.58	0.19	1.30	0.55	1.56	0.46	0.03
31.26	A3G3S2	1404.98	0.52	0.15	0.47	0.08	0.54	0.11	0.42	0.11	0.03
31.52	FA3G3S2	1477.90	0.57	0.23	0.66	0.22	0.76	0.36	0.86	0.33	0.04
34.59	A4G4S3	1155.80	0.83	0.14	0.83	0.24	1.01	0.19	0.94	0.35	0.01
